# Treatment of corn with lactic acid delayed *in vitro* ruminal degradation without compromising fermentation: a biological and morphological monitoring study

**DOI:** 10.3389/fvets.2024.1336800

**Published:** 2024-01-22

**Authors:** K. E. Tian, Gan Luo, Dicky Aldian, Masato Yayota

**Affiliations:** ^1^The United Graduate School of Agricultural Science, Gifu University, Gifu, Japan; ^2^College of Animal and Veterinary Science, Southwest University for Nationalities, Chengdu, China; ^3^Faculty of Applied Biological Sciences, Gifu University, Gifu, Japan; ^4^Education and Research Center for Food Animal Health, Gifu University, Gifu, Japan

**Keywords:** lactic acid, *in vitro* fermentation, starch, morphology, microflora

## Abstract

Grain processed by lactic acid (LA) is known to improve ruminant growth and health. However, the exact mechanism regarding rumen hydrolysis of LA-treated grain is still ambiguous. This experiment was designed to compare the effects of 5% LA treatment on the trophic and morphological variations in corn and to discover the alternations in ruminal hydrolysis between LA-treated and untreated corn macroscopically and microscopically using *in vitro* fermentation method. The results showed that, compared with untreated corn (CN), corn treated with 5% LA for 48 h (CNLA) experienced a decrease in the dry matter, albumin fraction, aNDFom, and water-soluble carbohydrate content but an increase in the resistant starch content. The *in vitro* fermentation showed that the pH of CNLA was higher, but dry matter disappearance was lower than that of CN. Most of the fermentation indices were unaffected, except for decreased *iso*-butyrate and *iso*-valerate. The abundances of total bacteria, *Prevotella* spp., *Streptococcus bovis*, and *Selenomonas ruminantium* were higher, but those of *Ruminococcus flavefaciens* and *Ruminococcus albus* were lower in CNLA than in CN. There were differences in the scanning electron micrographs between CNLA and CN after 3 h of fermentation. This study suggests that treating corn with LA for 48 h can induce changes in its nutrient composition and alter the bacterial flora during subsequent *in vitro* fermentation. These changes appeared to be crucial contributors to the beneficial effects observed in rumen fermentation.

## Introduction

Corn is the most widely produced grain worldwide (1) and serves as the primary energy substrate in intensive ruminant rearing systems in many countries (2–4). Compared to other cereal grains such as barley and sorghum, corn contains greater levels of starch, minerals, and vitamins (5, 6). However, the highly fermentable nature of corn can easily induce digestive disorders when applied to the diet at a high ratio, particularly in beef fattening industries where corn makes up more than 70% (and even beyond 90% in some cases) of the total mixed ration for maximizing beef production (7). An excessive amount of corn in the diet can drastically reduce ruminal pH, leading to body inflammation and hindering nutrient absorption in the hindgut, ultimately impacting ruminant growth performance (4, 8).

Numerous efforts have been made over the past few decades to improve corn utilization in ruminants through processing techniques. Lactic acid (LA) has long been used to improve starch productivity in various industries (9). After Zebeli’s and Ametaj’s laboratories introduced this method to dairy cattle and achieved beneficial results in enhanced milk fat production and reduced risk of inflammation (10, 11), the advantages of processing grains with LA have gradually been recognized by researchers. Compared to mechanical processing methods such as steam flaking and dry rolling, LA processing offers economic, safe, and operational benefits. Deckardt et al. (12) observed a lower amount of ruminal protozoa and enhanced fiber digestion *in vitro* when barley was treated with 1% LA. In a later experiment conducted by Yang et al. (8), beef cattle fed 1% LA-treated corn showed improved growth performance and reduced ruminal lipopolysaccharide (an inflammatory inducer) levels compared to cattle fed untreated corn. It was assumed that the advantage of LA-treated grain in ruminants can be attributed to the debranching effect of LA, which enables amylopectin to mimic the structure of amylose (13), since amylose has a slower digestion rate in the rumen that can lower the fermentation rate and increase ruminal pH (14). Additionally, LA treatment increased the ratio of resistant starch in the total starch content, allowing this portion of starch to escape ruminal digestion and be utilized in the intestine with the assistance of pancreatin (15). In previous studies, a reduction in the protein content of LA-treated grain has been observed (9, 13). However, the exact mechanism regarding ruminal hydrolysis of LA-treated grain is unclear. The insufficient knowledge regarding LA-treated grain nutrient variation and subsequent ruminal utilization has piqued our interest. We hypothesized that LA-treated grain can benefit rumen fermentation and alter bacterial flora via the manipulation effect of LA on grain nutrient components, and this effect would impact the *in vitro* fermentation process. Thus, the objective of this study was to investigate how LA-treated grain affects the nutrient value and rumen fermentation dynamics using corn as the test grain.

## Materials and methods

### Lactic acid treatment of corn

Treatment of corn was followed the procedures described in Deckardt et al. (12) and Martínez et al. (16). Briefly, corn was purchased from Toyohashi Feed Mills Co., Ltd. (Aichi, Japan), milled and screened through two consecutive sieves of 2.36 and 1.18 mm, and the particles retained on the 1.18 mm screen were recovered and steeped in tap water (CN) or 5% LA (DL-lactic acid, dissolved in tap water; CNLA) for 48 h. Then, corn particles were repeatedly and gently rinsed in flowing tap water until the pH reached 7.0.

### Ruminal inoculum and *in vitro* fermentation

Four healthy Japanese Shiba goats of similar body weight (37.1 ± 0.54 kg) and condition raised in the Yanagido farm at Gifu University were selected as ruminal fluid donors. All goats were previously maintained for 2 weeks by offering a 760 g forage diet [dry matter consisted of 7% alfalfa hay and 93% oats hay, according to NRC (17).] per day with *ad libitum* access to water and mineral block before sampling. On the sampling day, ruminal fluid was collected at 3 h after the morning feed using an esophageal tube following procedures described previously (18). The collected ruminal fluid was immediately strained through four layers of cheesecloth and mixed with preheated (39°C) buffer [the ruminal fluid to buffer ratio and buffer contents were detailed in Makkar et al. (19)] under continuous CO_2_ flushing (16, 20, 21). For differently treated corn (CN and CNLA) and fermentation time intervals (3, 6, 12, 18, and 24 h), 40 mL of the mixture and 0.5 g of corn particles were injected into a fermentation glass vial, flushed with CO_2_, sealed using a butyl rubber plug and tightened with an aluminum cap, as described in Deckardt et al. (12) and An et al. (20). Finally, vials were stabled on a rotary shaker water bath (140 rpm) and incubated at 39°C (16). The mixture without added corn particles was immediately stored at −80°C and used as the fermentation baseline (0 h). Glass vials for each fermentation time interval (3, 6, 12, 18, and 24 h) were immediately removed from the water bath on time, and terminated fermentation by chilling in ice water for 5 min. The incubation fluid (inoculant) was measured for pH using a pH meter (MP-220, Mettler-Toledo AG, Switzerland), collected into 2 mL tubes, and stored at −80°C for later analysis. The residual in the glass vials was rinsed in PBS (pH 7.2) three times and immediately used for morphological and microbial analysis.

### Chemical analysis

After treatment, corn was oven-dried at 60°C for 48 h and ground through a 0.5-mm screen for analysis of dry matter (DM; 934.01), crude protein (CP; 984.13), ash (942.05), ether extract (EE; 920.39) and acid detergent lignin (ADL; 973.18) according to the AOAC methods (22). The ADFom (acid detergent fiber expressed exclusive of residual ash) and aNDFom (neutral detergent fiber with heat-stable amylase and expressed exclusive of residual ash) were analyzed following the methods of Van Soest et al. (23). Cellulose was calculated as ADFom minus lignin (24). Non-fiber carbohydrates were calculated using the following formula: 100 − % aNDFom − % CP − % EE − % ash (25). Water soluble carbohydrates were measured by calculating reducing sugar content after anthrone reaction according to Koehler (26). Corn proteins were fractionated into albumin, globulin, zein, and glutelin, and concentrations were analyzed using bovine serum albumin (BSA) as the standard protein (27). For starch determination, treated corn was freeze-dried, ground to 0.5 mm and measured by comparison with standard D-glucose after incubation with α-amylase (heat stable; for total starch content), concanavalin A (for amylose content), and pancreatic α-amylase plus amyloglucosidase (for resistant starch content), according to instructions of the commercially available kits K-TSTA, K-AMYL, and K-RSTAR (Megazyme, Wicklow, Ireland).

For SCFA analysis, the inoculant was first centrifuged, then derivatized and extracted into a 1.5 mL sampling tube, and finally analyzed using UPLC-MS (Xevo^™^ QToF, Waters, Milford, MA) according to the method described in our previous study (28). The methane yield was calculated considering 90% hydrogen recovery according to Moss et al. (29), and the formula was CH_4_ (mmol/L) = 0.45 × acetate (mmol/L) − 0.275 × propionate (mmol/L) + 0.4 × butyrate (mmol/L). Ammonia nitrogen was determined by the phenol-hypochlorite method (30) and digitized using an automatic microplate reader.

### Microbial analysis

Microbial DNA was extracted from 0.5 mL of homogenized residual according to Yu and Morrison (31) and purity was determined with a spectrophotometer. Extracted DNA was diluted to 10 ng/μL, and absolute quantification was performed using qPCR with specific primers of each targeted bacteria (Supplementary Table S1) and qPCR SYBR Mix (Toyobo, Osaka, Japan), the selected bacteria, including *Prevotella* spp., *Prevotella ruminicola* (*P. ruminicola*), *Streptococcus bovis* (*S. bovis*), *Selenomonas ruminantium* (*S. ruminantium*), *Ruminococcus flavefaciens* (*R. flavefaciens*), *Ruminococcus albus* (*R. albus*) and *Butyrivibrio fibrisolvens* (*B. fibrisolvens*) were the most pivotal rumen bacteria involved in the rumen hydrolyzing process (31–33). Later, quantified DNA was purified, and a standard curve was established for each targeted microbe. Estimation of gene copy numbers for selected bacteria were log-transformed and presented as gene copy numbers per mL of fermentation fluid. The activity of bacterial enzymes, including α-amylase, cellulase, protease, and lipase, were measured by quantification of reducing components released from culture digestion of each related substrate (34), and enzyme activities were defined as the amount of enzyme required to release 1 μmol of reducing components/min (or h) per mL of fermentation fluid.

### Morphological analysis

The morphological structure of corn particles and fermentation residuals were viewed by scanning electron microscopy (SEM). The SEM procedures per treatment were as follows: (i) the particles were fixed in the 1st phosphate buffer (0.2 M; containing 0.5% glutaraldehyde and 0.15% ruthenium red, pH 7.2) for 2 h. (ii) The particles were transferred into the 2nd phosphate buffer (0.2 M; containing 5% glutaraldehyde and 0.05% ruthenium red, pH 7.2) for 2 h. (iii) The buffer was removed, and the samples were washed using 3rd phosphate buffer (0.2 M; containing 0.05% ruthenium red, pH 7.2) three times with 20 min interval stands. (iv) Samples were dehydrated using increasing concentrations of ethanol (10, 20, 30 50, 70, 90, and 99.5%) with intervals of 15 min (16, 35). After pretreatment, the corn particles were freeze-dried, stuck on aluminum stubs, sputter coated with osmium, and viewed by SEM (S-4800, Hitachi, Tokyo, Japan).

### Statistical analysis

All data were analyzed using the MIXED procedure of SPSS (v20.0, IBM, Armonk, NY, United States) according to the following model:


$$Y_{ijl}=\mu +\beta_{i}+T_{j}+D_{l}+{\left(TD\right)}_{jl}+e_{ijl}$$

where *Y_ijl_* represents the dependent variable, *μ* represents the overall mean, *β_i_* represents the random effect of ruminal fluid held within a bottle, *T_j_* represents the fixed effect of measurement time, *D_l_* represents the fixed effect of grains, (*TD*)*_jl_* represents the grain by time interaction and *e_ijl_* represents the random residual effect. Differences in the means were compared by *t*-test, and a *p*-value lower than 0.05 was considered significant. Measurement of nutrients in LA-treated and untreated corn was conducted during lab work, and results were presented as the mean ± SD. All indices were measured quadruplicate.

## Results

### Nutrient components and morphological structure of corn

**Figure 1:**
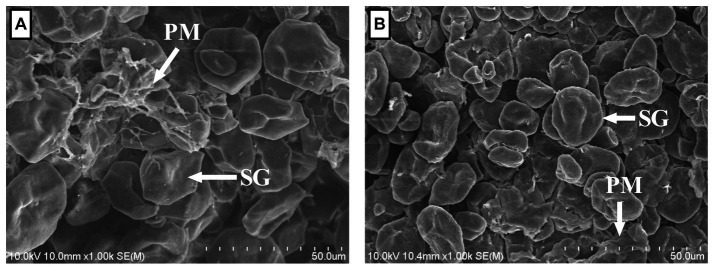
Scanning electron micrographs of corn steeped with tap water for 48 h **(A)** and corn steeped with 5% lactic acid (dissolved in tap water) for 48 h **(B)** at 1000× focal length; SG, starch granule; PM, proteinaceous matrix.

As shown in Table 1, the DM, water-soluble carbohydrate, aNDFom, and fractionated albumin of CNLA were lower than those of CN. Conversely, the resistant starch content in CNLA was greater than that in CN. However, processing corn with LA seemed to result in little difference in the content of CP, EE, ADFom, ADL, cellulose, starch, or amylose. The morphological structure of corns were shown in Figure 1. The most evident variation induced by LA processing is the reduction in the proteinaceous matrix that surrounded starch granules on CNLA compared with CN. However, there was little noticeable difference in the morphological structure of starch granules to be reported between CN and CNLA.

**Table 1:** Nutrient composition of corn and corn steeped with lactic acid (% of dry matter basis; presented as the mean with standard deviation from quadruplicate measurements).

Items	CN	CNLA	*p*-value
Dry matter	76.64 ± 0.09	74.40 ± 0.09	<0.01
Crude protein	7.48 ± 0.20	7.25 ± 0.13	0.14
Albumin^a^	1.50 ± 0.09	0.99 ± 0.11	<0.01
Globulin^a^	1.15 ± 0.14	0.89 ± 0.15	0.07
Zein^a^	3.58 ± 0.24	3.62 ± 0.24	0.87
Glutelin^a^	2.61 ± 0.17	2.70 ± 0.16	0.50
Ether extract	1.79 ± 0.09	1.80 ± 0.02	0.86
aNDFom^b^	8.78 ± 0.20	8.41 ± 0.14	0.04
ADFom	2.56 ± 0.16	2.34 ± 0.18	0.16
ADL	1.22 ± 0.19	0.99 ± 0.12	0.12
Cellulose	1.34 ± 0.03	1.35 ± 0.06	0.80
Ash	3.42 ± 0.19	3.66 ± 0.17	0.13
Starch	66.16 ± 0.65	66.49 ± 0.74	0.59
Amylose	21.95 ± 1.43	22.15 ± 0.32	0.82
Resistant starch	2.29 ± 0.20	8.08 ± 0.13	<0.01
Non-fiber carbohydrates	78.63 ± 0.88	78.78 ± 1.03	0.85
Water soluble carbohydrates	0.65 ± 0.02	0.52 ± 0.03	<0.01

a Protein content equivalents to bovine serum albumin.

b aNDFom, neutral detergent fiber with heat-stable amylase and expressed exclusive of residual ash; ADFom, acid detergent fiber expressed exclusive of residual ash; ADL, acid detergent lignin.

### Variations in *in vitro* fermentation

**Figure 2:**
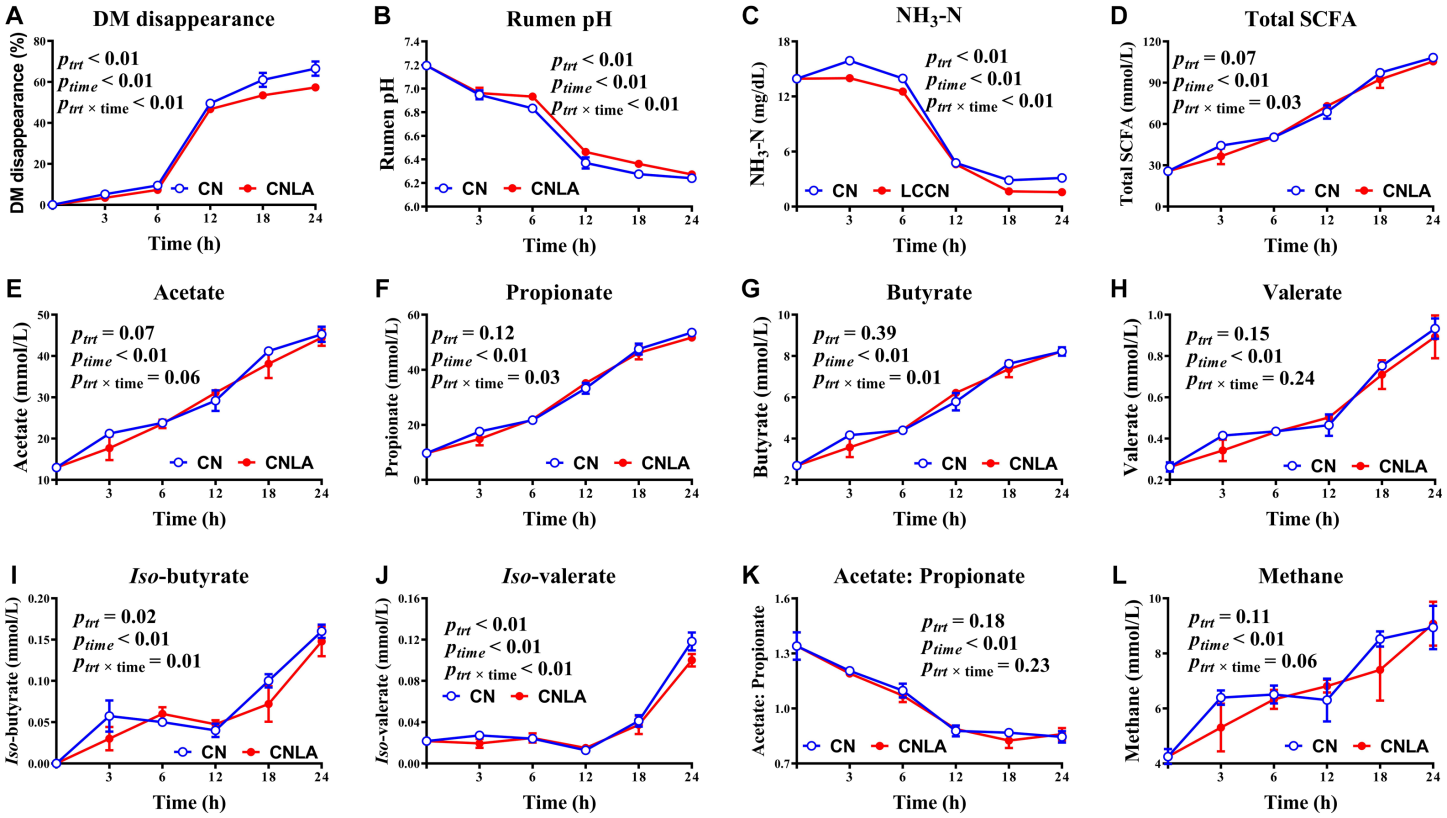
Fermentation characteristics including DM disappearance **(A)**, rumen pH **(B)**, ammonia nitrogen **(C)**, total short-chain fatty acid **(D)**, acetate **(E)**, propionate **(F)**, butyrate **(G)**, valerate **(H)**, *iso*-butyrate **(I)**, *iso*-valerate **(J)**, acetate to propionate ratio **(K)** and methane **(L)** as affected by corn treatment (*p*_trt_), fermentation time (*p*_time_), and their interactions (*p*_trt × time_). Lines marked with blue open circles represent the treatment of corn steeped with tap water for 48 h (CN), and lines marked with red closed circles represent the treatment of corn steeped with 5% lactic acid (dissolved in tap water) for 48 h (CNLA).

In the present study, DM disappearance was greater in CN than in CNLA (*p* < 0.01, Figure 2), and the magnitude of the decrease became more pronounced at 18 and 24 h. The pH of CNLA was greater than that of CN (*p* < 0.01), especially at 6, 12, and 18 h. The concentration of NH_3_-N in CNLA was lower than that in CN (*p* < 0.01), and the difference was evident during the whole process of fermentation except at 12 h. The molar proportions of *iso-*butyrate and *iso-*valerate were significant between CN and CNLA (*p* < 0.01), but the difference was also dependent on time. The content of total SCFA, acetate, propionate, butyrate, valerate, acetate to propionate ratio, and calculated methane production were not influenced by LA processing (*p* > 0.05). The time effect was significant for all indices measured between CN and CNLA in this section (*p* < 0.01). The interaction between LA treatment and incubation time was significant for all indices except for acetate, valerate, acetate to propionate ratio, and methane production (*p* > 0.05).

**Figure 3:**
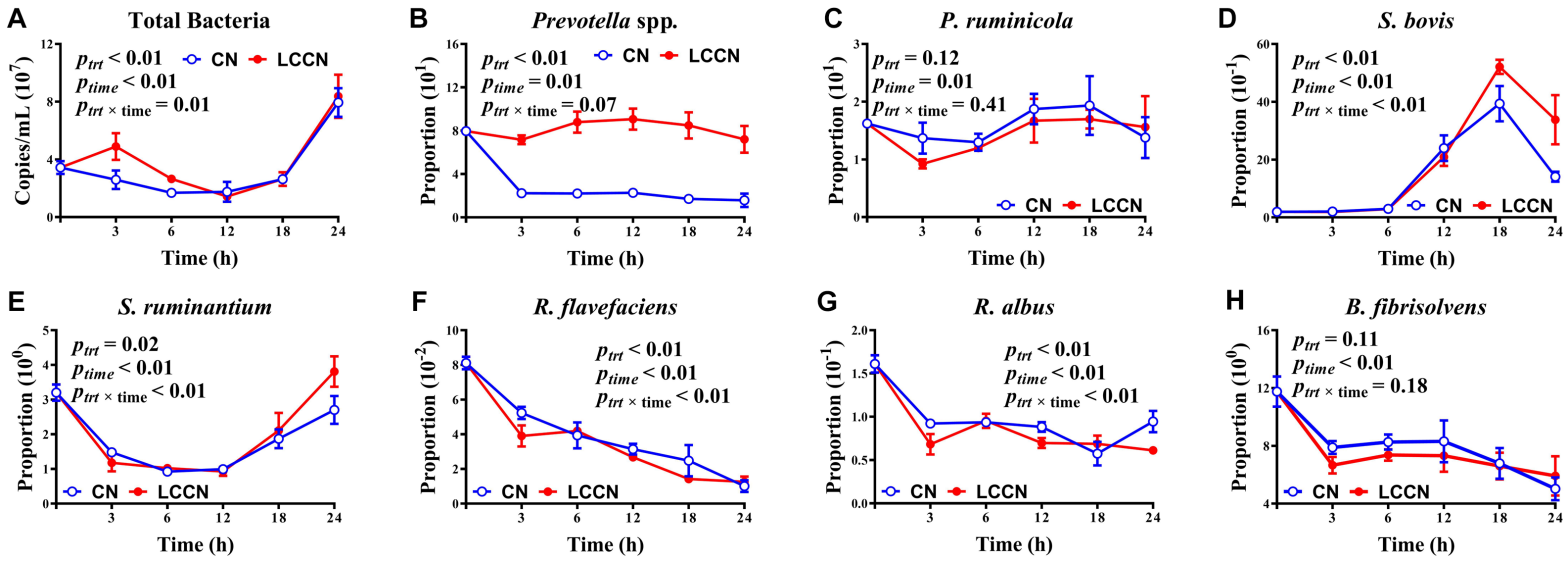
Effects of corn treatment (*p*_trt_), fermentation time (*p*_time_), and their interactions (*p*_trt × time_) on the absolute abundance of total bacteria **(A)** and specific bacterial populations including *Prevotella* spp. **(B)**, *Prevotella ruminicola* **(C)**, *Streptococcus bovis* **(D)**, *Selenomonas ruminantium* **(E)**, *Ruminococcus flavefaciens* **(F)**, *Ruminococcus albus* **(G)** and *Butyrivibrio fibrisolvens* **(H)** (% of total bacterial 16S rDNA). Lines marked with blue open circles represent the treatment of corn steeped with tap water for 48 h (CN), and lines marked with red closed circles represent the treatment of corn steeped with 5% lactic acid (dissolved in tap water) for 48 h (CNLA).

LA treatment of corn increased the absolute abundance of total bacteria (*p* < 0.01, Figure 3), and the effect was more obvious at 3 h and 6 h of fermentation. The proportion of *Prevotella* spp. was significantly greater in CNLA than in CN (*p* < 0.01). The proportion of *S. bovis* was greater in CNLA than in CN, especially at 18 h and 24 h. The proportion of *S. ruminantium* was greater in CNLA than in CN (*p* = 0.02), but that of *R. flavefaciens* was greater in CN than in CNLA (*p* < 0.01), and the difference in these two bacterial species between CN and CNLA was obvious at 24 h and 3 h of fermentation, respectively. The treatment effect was significant for *R. albus* proportion (*p* < 0.01), but the proportion was greater at 3 h and 12 h, and lower at 24 h in CN than in CNLA. The proportion of *P. ruminicola* and *B. fibrisolvens* received little disturbance from corn treatment (*p* = 0.11). The time effect was significant for all identified microbes (*p* < 0.01). The interactive effect of LA treatment and fermentation time was significant for total bacteria, *S. bovis*, *S. ruminantium*, *R. flavefaciens*, and *R. albus* (*p* < 0.01) but not for *Prevotella* spp., *P. ruminicola* or *B. fibrisolvens* (*p* > 0.05).

**Figure 4:**
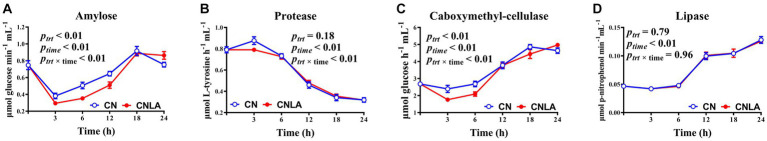
Diurnal variation in α-amylase **(A)**, protease **(B)**, Carboxymethyl-cellulase **(C)**, and lipase **(D)** activities as affected by corn treatment (*p*_trt_), fermentation time (*p*_time_), and their interactions (*p*_trt × time_). Lines marked with blue open circles represent the treatment of corn steeped with tap water for 48 h (CN), and lines marked with red closed circles represent the treatment of corn steeped with 5% lactic acid (dissolved in tap water) for 48 h (CNLA).

The enzyme activities of amylose and carboxymethyl-cellulase were lower in CNLA than in CN (*p* < 0.01, Figure 4), but the difference varied with time. However, protease and lipase activity were not significant under LA treatment (*p* > 0.05). The time effect was significant for all the enzymes. The treatment × time interaction was significant for amylose, protease, and carboxymethyl-cellulase (*p* < 0.01) but not for lipase (*p* > 0.05).

### Morphological structure of corn grains during fermentation

The white, protein-like cover on the surface of corn before fermentation and the “reticular” matrix after fermentation observed by SEM were all defined as “proteinaceous matrix (PM)” in the following text, similar to the research of Martínez et al. (16), and will be further explained in the discussion section.

**Figure 5:**
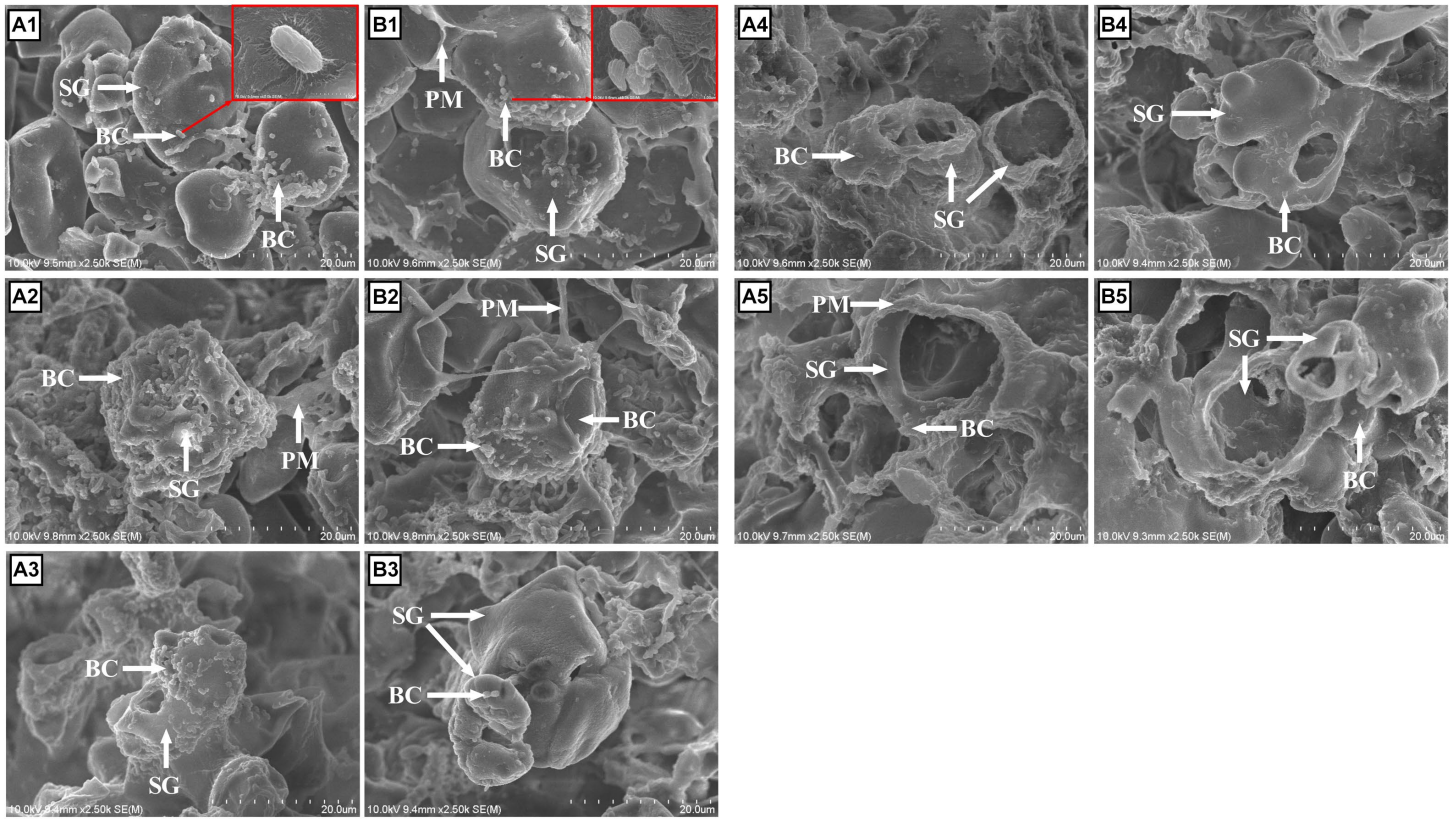
Morphological features of corn steeped with tap water for 48 h (CN; **A**), and corn steeped with 5% lactic acid (dissolved in tap water) for 48 h (CNLA; **B**) viewed by scanning electron microscopy at 3 **(A1, B1)**, 6 **(A2, B2)**, 12 **(A3, B3)**, 18 **(A4, B4)**, and 24 **(A5, B5)** hours of in vitro fermentation at 2500x of focal length. BC, bacterial cells; SG, starch granule; PM, proteinaceous matrix.

The changes in the ultrastructures of corn and LA-treated corn were noticeable after 3 h of fermentation. At 3 h, hydrolysis of the PM on the surface was very rapid compared with the starch granule (SG), so the PM nearly disappeared on the CN surface, and only a small part of PM could be visualized on the CNLA. Instead, clustered bacterial cells began to colonize the SG, with a greater amount on the CN compared with CNLA (Figures 5A1,B1; larger size figures were deposited in Supplementary Figure S1). After 6 h, no PM was observed on the surface of CN and CNLA, and the SG of CN collapsed with total bacterial colonization. There was also a considerable amount of bacteria on the SG of the CNLA, but the shape of the SG was clearly maintained (Figures 5A2,B2). At 12 h, the bacterial amount on the SG surface decreased, and cavities began to appear on the SG. The CN was more digested than CNLA because of the larger diameter of the cavities (Figures 5A3,B3). After 18 h, the cavities on the starch granule were even larger. The inner SG substrate of CN was nearly digested. However, the SG of the CNLA still persisted at a large part (Figures 5A4,B4). Finally, at 24 h, only a limited part of the starch granule was left. However, the CNLA starch granule substrate was still preserved more than CN (Figures 5A5,B5).

## Discussion

### Effect of LA treatment on corn nutrients and morphological structure

LA treatment of cereal grains affects ruminal fermentation primarily by modifying their nutritional properties (10). In the present study, we observed significant alterations in both the ultrastructure and nutrient compositions of corn by LA treatment, with notable changes occurring in the content of albumin and resistant starch. Regarding corn ultrastructure, the most prominent change induced by LA treatment was the reduction in the PM on the CNLA compared to CN. This observation aligns with Dailey et al. (9), who reported protein leakage into the steeping solution during 0.5% LA treatment. Surprisingly, despite the structural change, there was little difference in CP content between CN and CNLA; however, the albumin fraction was lower in CNLA than in CN. Vötterl et al. (13) indicated that the CP content of grains can vary with the duration of LA treatment. In their study, the CP proportion of corn treated with 25 g/kg LA was 8.93% at 0 h, increased to 9.14% at 24 h, but decreased to 8.78% at 48 h. Serna-Saldívar and Mezo-Villanueva (36). also highlighted the potential of LA treatment to increase soluble protein levels, particularly albumin and globulin in the steeping solution. Thus, it is reasonable to conclude that the 5% LA treatment can alter albumin fractions, but the effect was not strong enough to induce variation in the CP content. The lower aNDFom content in CNLA to CN was consistent with the result of Vötterl et al. (13), which might be caused by either LA hydrolysis of glycosidic bonds in the hemicellulose fractions (9) or LA activation of hemicellulase, including xylanase and mannanase (13) in the corn.

Regarding the carbohydrate content, previous study have suggested that LA treatment of barley can increase the amylose content due to the linearization effect of LA, which enables amylopectin to mimic the structure of amylose (37), but the similar content of amylose observed between CN and CNLA implied that this was not the case in the present study. A plausible explanation for this consequence was that the amylopectin content was generally lower in corn than in barley (14) that attenuated the manipulation effect of LA on this nutrients. Collectively, the decrease in DM could be the cumulative effect (13, 37) of reduction of albumin, aNDFom, and water-soluble carbohydrate.

### Effect of LA treatment on *in vitro* fermentation parameters

The original purpose of LA treatment of grains was to shift starch fermentation from the rumen to the hindgut (38). While unexpected benefits, such as an elevated ruminal pH, were reported during animal trials (10, 11). The lower DM disappearance in CNLA to CN in our study can be attributed to the greater resistant starch content of CNLA compared with CN, which can also be proved by the remaining starch granules on the micrographs of CNLA compared with CN at 24 h. On the other hand, it was suggested that intensive rumen fermentation occurs mainly from 0 to 12 h postfeeding (39). Thus, the lower DM disappearance in CNLA to CN, especially at 18 h and 24 h of fermentation, can be attributed to the greater content of resistant starch in CNLA because this type of starch generally degrades more slowly (or not degrades) in the rumen than other starch types. The pH-elevating effect of LA treatment was in accordance with the study of Iqbal et al. (11), in which 1% LA-treated barley was fed to dairy cows. However, the beneficial effect on pH cannot be simply attributed to the reduced albumin fraction, since the degradation rate of CP in the rumen is often greater than that of starch (40), and CP is generally exhausted at approximately 12 h of fermentation (6). Therefore, the more highly resistant starch in CNLA than CN is also suggested to contribute to the pH elevation.

Contrary to the result that CNLA showed a lower DM disappearance, the total SCFA in CNLA and CN was not affected by LA treatment. Iqbal et al. (10) also reported no effect of 0.5% LA-treated barley on total ruminal SCFA but did observe lower total SCFA levels at 2 and 4 h of fermentation. Partially in agreement with their report, we noticed that LA treatment did not provoke any difference in total SCFA between CN and CNLA throughout the experiment. Rumen SCFA mainly originate from the microbial fermentation of carbohydrates (41). Therefore, the discrepancy in total SCFA levels at 2 to 4 h of fermentation in their study can be attributed to the lower content of soluble carbohydrates (an 8% decrease) induced by LA treatment compared to the similar soluble carbohydrate content between CN and CNLA in our study (0.65% of DM in untreated corn compared to 0.52% in LA-treated corn). The lower branched-chain SCFA, together with lower NH_3_-N, indicate reduced protein utilization in CNLA compared to CN. However, this contrasts with the similar CP content between CNLA and CN. In addition, the lower volume of albumin was insufficient to suppress the ammonia concentration for the entire 24 h. Although Harder et al. (32) suggested that LA treatment might protect CP from degradation, the difference in NH_3_-N between CN and CNLA observed at 18 h and 24 h of fermentation remain to be explained.

### Effect of LA treatment on bacterial flora, enzyme activities, and morphological structure of cereals during fermentation

In accordance with Harder et al. (42), who treated barley with 5% LA, we noticed a similar increase in total bacteria in CNLA against CN until 6 h of fermentation. However, the result disagreed with that of Metzler-Zebeli et al. (33), who used 1% LA-treated barley and observed no difference in total bacteria throughout the fermentation process. Khafipour et al. (43) suggested that lower pH will decrease the abundance of total bacteria. Thus, it can be suggested that the protective effect of LA on CP (32) prevented a drastic drop in pH, thereby allowing more bacteria to survive during 0 to 6 h. Notably, the proportion of *Prevotella* spp. markedly differed between CN and CNLA throughout the experiment. *Prevotella* spp. is one of the most abundant genera that exists in grain-feeding ruminants and offers ruminants a high-grain diet that elevates the abundance of this bacterial genus, thus increasing the production of propionate (43, 44). However, the propionate level in our study was not affected by LA treatment. Rather, it was more appropriate to infer that *Prevotella* spp. is also sensitive to the increase in resistant starch (42). However, the unaffected proportion of *P. ruminicola* (generally accounting for a large proportion of *Prevotella* spp. (45),) indicated that the response of this genus to LA treatment still needs further research. The increased proportion of *S. bovis* and *S. ruminantium* at 18 h and 24 h, respectively, are probably due to their capacity to degrade resistant starch (46). The decrease in *R. flavefaciens* and *R. albus* proportion by LA treatment might be the response to lower aNDFom content, as both microbes actively participate in ruminal fiber degradation (47). Regarding enzyme activities, the decreased amylose can possibly be attributed to the lower proportion of *P. ruminicola*, which is a well-known ruminal amylolytic bacteria and has versatility in carbohydrate utilization (48). Likewise, the reduction in cellulose activity might account for the lower proportion of *R. flavefaciens* and *R. albus* due to their involvement in fiber digestion (47). The lower activity of the protease protective effect of LA can be partly attributed to the protective effect of LA on CP (as mentioned above). Seemingly, the selected bacterial abundance and fermentation indices were insufficient to explain the unaffected lipase activity. A previous study showed high lipase activity when protozoa attached to feed particles (49). Protozoa abundance was not analyzed in our study but remained stable in Metzler-Zebeli et al. (33), who used 1% LA to treat barley. Furthermore, there was also a report indicating that *B. fibrisolvens* [proportion was not affected by LA treatment in our study and Mickdam et al. (44)] has the potential to utilize lipids (50). Thus, the unaffected proportion of *B. fibrisolvens* might stabilize the lipase activity between CN and CNLA.

The noticeable differences in morphological structure between CN and CNLA were considered to result from multiple factors. Generally, the fermentation rate of nutrients follows the order of protein > starch > fiber (6, 51, 52). Therefore, differences in the morphological structure of cereals can be better elucidated by considering the fermentation process as distinct stages, which encompass protein (3 to 6 h), starch (6 to 12 h), and fiber (18 to 24 h) fermentation. These stages, while interleaved, exhibit unique fermentation characteristics. Firstly, the fast fermentation of protein led to a larger amount of bacterial cell (BC) observed on the morphological structure of CN compared to CNLA from 3 to 6 h. This can be attributed to the higher albumin content that nourished bacteria specifically fed on proteins. Subsequently, with the onset of starch fermentation, the differences in morphological structure from 6 to 12 h can be attributed to the lower pH continuing from protein fermentation, facilitating the fermentation of SG in CN (53), or higher RS content restricting the fermentation of SG in CNLA. Furthermore, the absence of a pH difference from 18 h may be attributed to the nearly complete starch fermentation and the initiation of fiber fermentation (16). While some previous studies indicated that LA treatment could reduce NDF content, the relatively low fiber content in cereal grains and its limited contribution to fermentation, such as pH regulation (54), appear to constrain the impact of LA treatment on morphological structures during 18 to 24 h. Therefore, despite the differences observed in SG, there was little noticeable distinction in PM between CN and CNLA.

Regarding the term “proteinaceous matrix” used throughout our study for explaining the SEM results, it is worth noting that research related to this topic is limited. However, we believe that this term was suitable for describing the “white cover” on the starch surface at 0 h and the “reticular matrix” until 12 h, given the relatively high CP content remaining in the corn particles during this period. Nevertheless, when we compare our observations with those of Martínez et al. (16), we raise some doubts as to whether the “reticular matrix” observed after 12 h can truly be termed a “proteinaceous matrix” due to general exhaustion of CP at approximately 12 h of rumen fermentation (6). This coincides with the decreased NH_3_-N content followed by bacterial fermentation of CP. Speculatively, the term “fibrous matrix (elevated activity of carboxymethyl-cellulase)” or “pectin matrix (main component of cell wall)” might be more suitable when describing the structure after 12 h of fermentation.

Besides straightly treating cereal grains with LA, many studies investigated the effects of treating cereal grains with lactic acid bacteria (LAB), a bacterial species widely employed in silage preservation by feeding industries (55, 56). In contrast to the protective effect of LA on cereal amino acids, LAB treatment of cereal can result in increased concentrations of available amino acids (57), thus facilitating their utilization in the rumen. Moreover, LAB treatment has been reported to offer comparable advantages to LA treatment in enhancing ruminant mineral utilization. This is crucial since approximately 60% to 80% of cereal minerals exist in the form of cation-salt chelates (58), particularly phytate phosphorus within plants, restricting their availability for ruminants (59). Several studies have highlighted the capabilities of both LA and LAB to release minerals, such as calcium (Ca) and phosphorus (P), from such chelates (13, 32, 60). It is important to note that LAB bacteria, such as *Lactobacilli* spp. are predominant cereal degraders (35), yet few studies evaluated whether the pretreatment of cereals with LAB may cause the loss of cereal nutrients before feeding to ruminants. A comprehensive evaluation of LAB is still needed before large application in practical production.

## Conclusion

Taken together, the results of this study and the consistency of our *in vitro* observations with previous *in vivo* research underscores the strong representativeness of our data for actual ruminant responses. On the other hand, the greater pH observed in CNLA compared to CN confirmed our hypothesis that the enhancement in resistant starch and decrease in albumin and the subsequent alternations in bacterial flora during fermentation play a pivotal role in the beneficial effects of LA-treated corn on rumen fermentation. Additionally, we provided the first visual description of the ultrastructural changes in both LA-treated and untreated corn during a 24 h fermentation period, offering a valuable reference for future in-depth investigations.

## Data availability statement

The original contributions presented in the study are included in the article/Supplementary material, further inquiries can be directed to the corresponding author.

## Ethics statement

This study was approved by the Committee for Animal Research and Welfare of Gifu University (Approval ID: #AG-P-N-20230048). All animal experimental procedures were conducted following the Guidelines for Proper Conduct of Animal Experiments (Science Council of Japan, 2006) and the Guidelines of Animal Research and Welfare of Gifu University (2008).

## Author contributions

KT: Conceptualization, Formal analysis, Investigation, Methodology, Writing – original draft, Writing – review & editing. GL: Conceptualization, Methodology, Writing – review & editing. DA: Investigation, Methodology, Writing – review & editing. MY: Funding acquisition, Project administration, Supervision, Writing – review & editing.
