# Supplementary material for: Treatment of corn with lactic acid delayed in vitro ruminal degradation without compromising fermentation: a biological and morphological monitoring study
Source: Front Vet Sci. 2024 Jan 22;11:1336800. doi: 10.3389/fvets.2024.1336800 (PMC10839040; doi:10.3389/fvets.2024.1336800)
Supplement: Supplementary file 1 [file Data_Sheet_1.pdf]

Supplementary Table

List of primers for real-time PCR assays.

Target microbes	Primers (5′–3′)[1]	Length (bp)	References
Total bacteria[2]	F: CGGCAACGAGCGCAACCC	130	Denman and McSweeney (2006)
	R: CCATTGTAGCACGTGTGTAGCC		
Ruminococcus flavefaciens	F: CGAACGGAGATAATTTGAGTTTACTTAGG	132	Denman and McSweeney (2006)
	R: CGGTCTCTGTATGTTATGAGGTATTACC		
Prevotella spp.	F: GGTTCTGAGAGGAAGGTCCCC	121	Bekele et al. (2010)
	R: TCCTGCACGCTACTTGGCTG		
Prevotella ruminicola	F: GAAAGTCGGATTAATGCTCTATGTTG	74	Stevenson and Weimer (2007)
	R: CATCCTATAGCGGTAAACCTTTGG		
Selenomonas ruminantium	F: CAATAAGCATTCCGCCTGGG	138	Stevenson and Weimer (2007)
	R: TTCACTCAATGTCAAGCCCTGG		
Streptococcus bovis	F: TTCCTAGAGATAGGAAGTTTCTTCGG	127	Stevenson and Weimer (2007)
	R: ATGATGGCAACTAACAATAGGGGT		
Butyrivibrio fibrisolvens	F: TAACATGAGTTTGATCCTGGCTC	113	Yang (2007)
	R: CGTTACTCACCCGTCCGC		
Ruminococcus albus	F: GTTTTAGGATTGTAAACCTCTGTCTT	273	Yang (2007)
	R: CCTAATATCTACGCATTTCACCGC		

[1] Primers were assembled from Integrated DNA Technologies, Inc. (Tokyo, Japan).

[2] The qPCR conditions for total bacteria and *R. flavefaciens* were as follows: one cycle of 50 °C for 2 min and 95 °C for 2 min for initial denaturation and 40 cycles of 95 °C for 15 s and 60 °C for 1 min for primer annealing.

The qPCR conditions for *Prevotella* spp. were as follows: denaturation at 95 °C for 10 min, followed by 40 cycles of 95 °C for 15 s, annealing at 55 °C for 5 s and 72 °C for 22 s.

The qPCR conditions for *P. ruminicola* and *S. ruminantium*, *S. bovis*, were as follows: initial hold for 10 min followed by 50 cycles of 95 °C for

15 s and 61 °C for 90 s for annealing.

The qPCR conditions for *B. fibrisolvens* and *R. albus* were as follows: initial hold at 50 °C for 2 min and 95 °C for 10 min for initial denaturation, followed by 40 cycles of 95 °C for 15 s and 60 °C for 1 min for primer annealing.

[3] References for Supplementary Table

Denman, S.E., McSweeney, C.S., 2006. Development of a real-time PCR assay for monitoring anaerobic fungal and cellulolytic bacterial populations within the rumen. FEMS Microbiology Ecology 58, 572–582.

Bekele, A.Z., Koike, S., Kobayashi. Y., 2010. Genetic diversity and diet specificity of ruminal *Prevotella* revealed by 16S rRNA gene-based analysis. FEMS Microbiology Letters 305, 49–57.

Stevenson, D.M., Weimer, P.J., 2007. Dominance of *Prevotella* and low abundance of classical ruminal bacterial species in the bovine rumen revealed by relative quantification real-time PCR. Applied Microbiology and Biotechnology 75, 165–174.

Yang, S., 2007. Effects of soybean oil and linseed oil supplementation on population of ruminal bacteria and fermentation parameters in dairy cows. PhD thesis, Chinese Academy of Agricultural Sciences, Beijing, China.

Supplementary Figure

Morphological features of corn steeped with tap water for 48 h (A1), and corn steeped with 5% lactic acid (dissolved in tap water) for 48 h (B1) viewed at 1000x of focal length, fermentation at 3 (A2, B2), 6 (A3, B3), 12 (A4, B4), 18 (A5, B5) and 24 (A6, B6) hours viewed at 2500x of focal length, and bacterial cells appeared on the untreated corn (A7) and LA treated corn (B7) surface at 3 h of fermentation viewed at 40,000x of focal length.

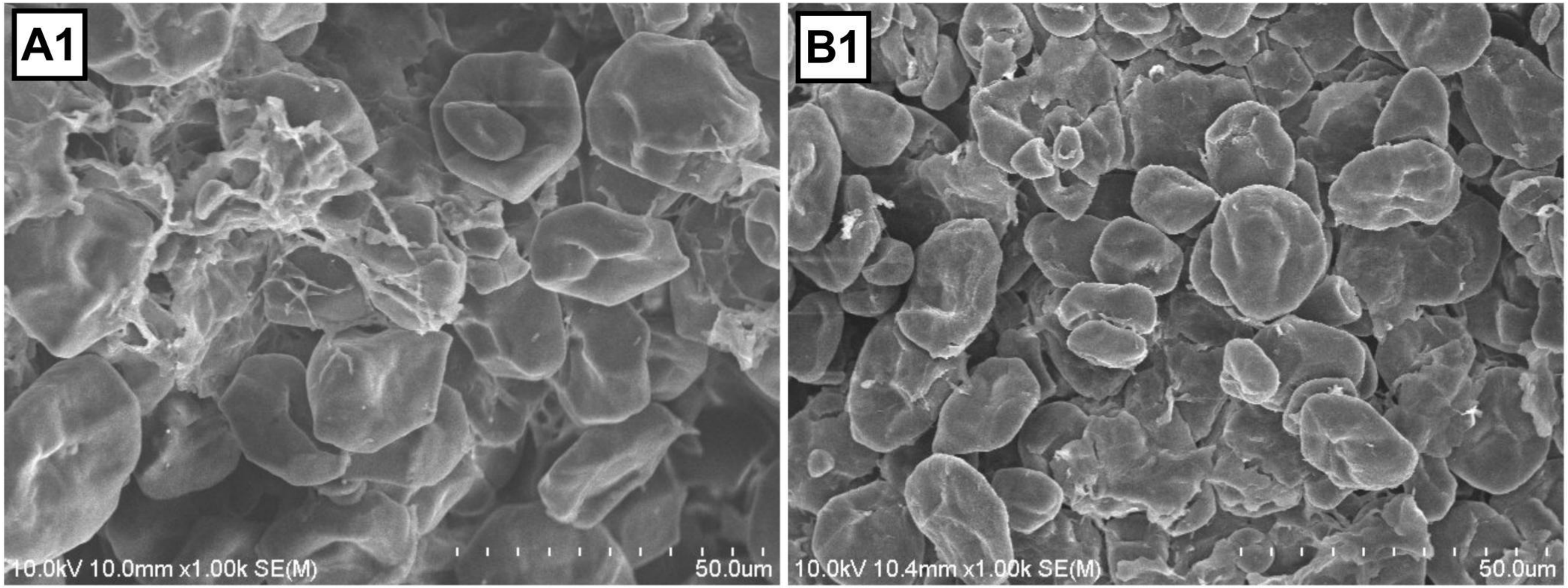

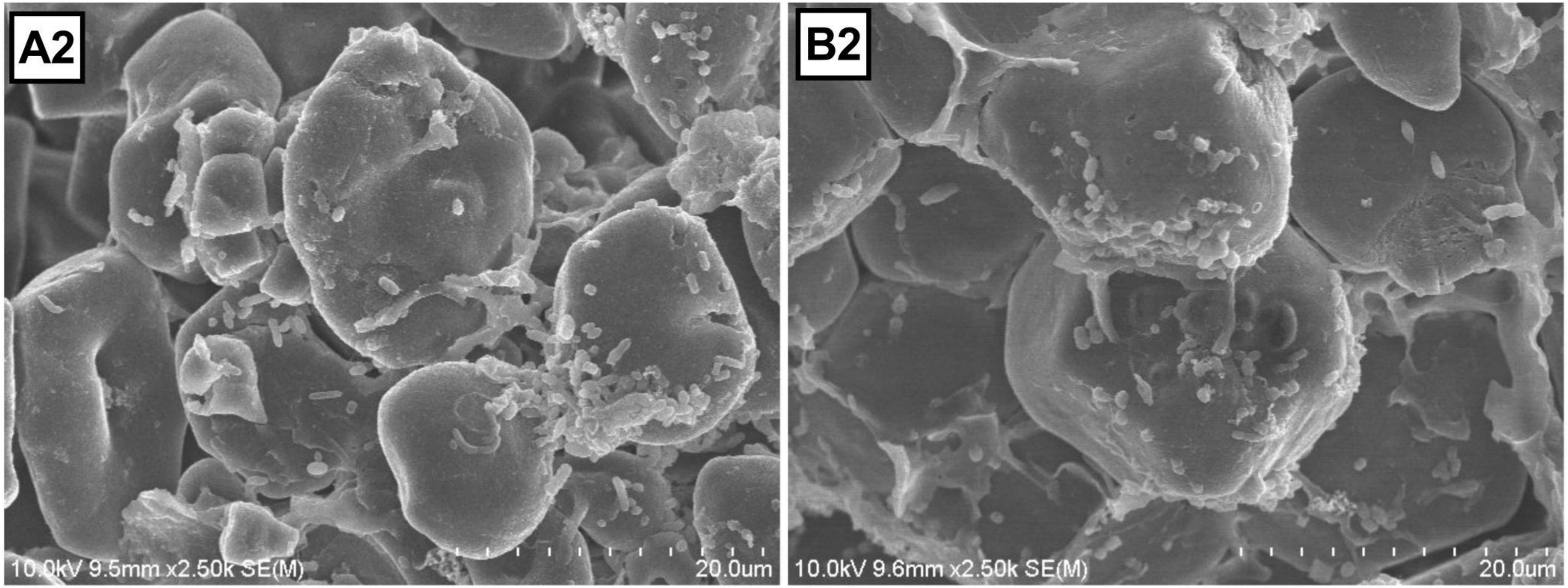
A2
10.0kV 9.5mm x2.50k SE(M)
20.0um
B2
10.0kV 9.6mm x2.50k SE(M)
20.0um

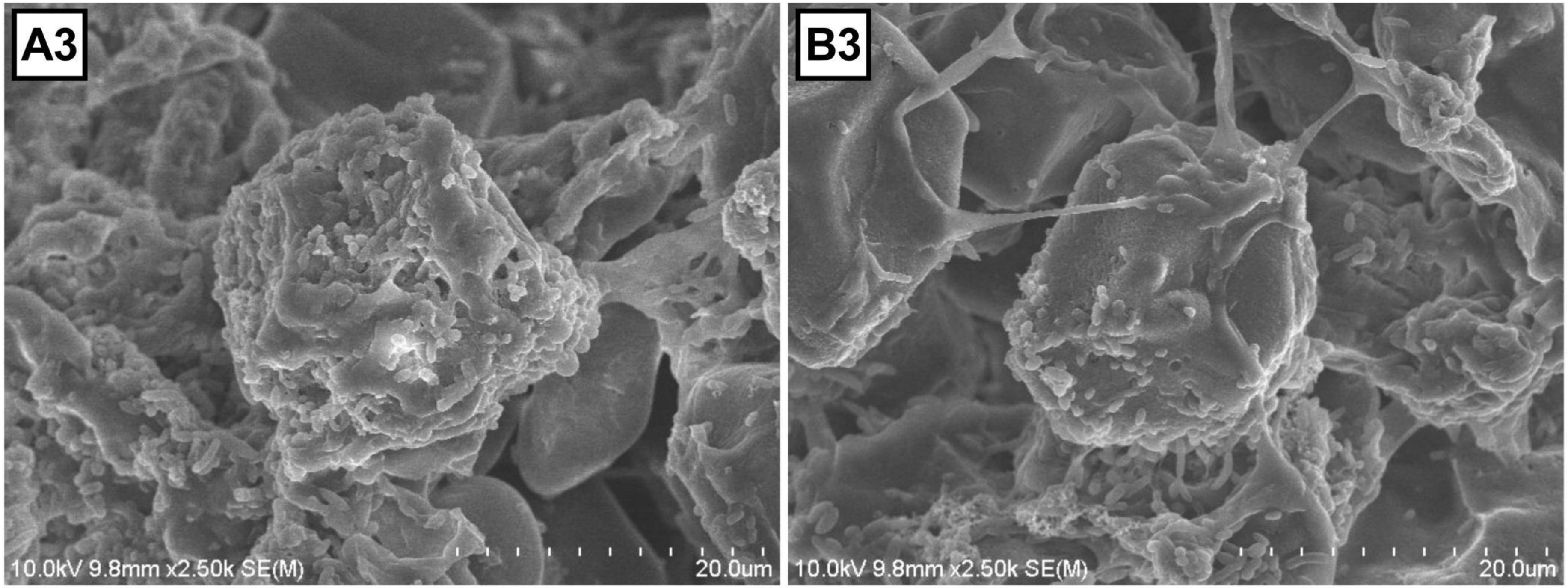
A3
10.0kV 9.8mm x2.50k SE(M)
20.0um
B3
10.0kV 9.8mm x2.50k SE(M)
20.0um

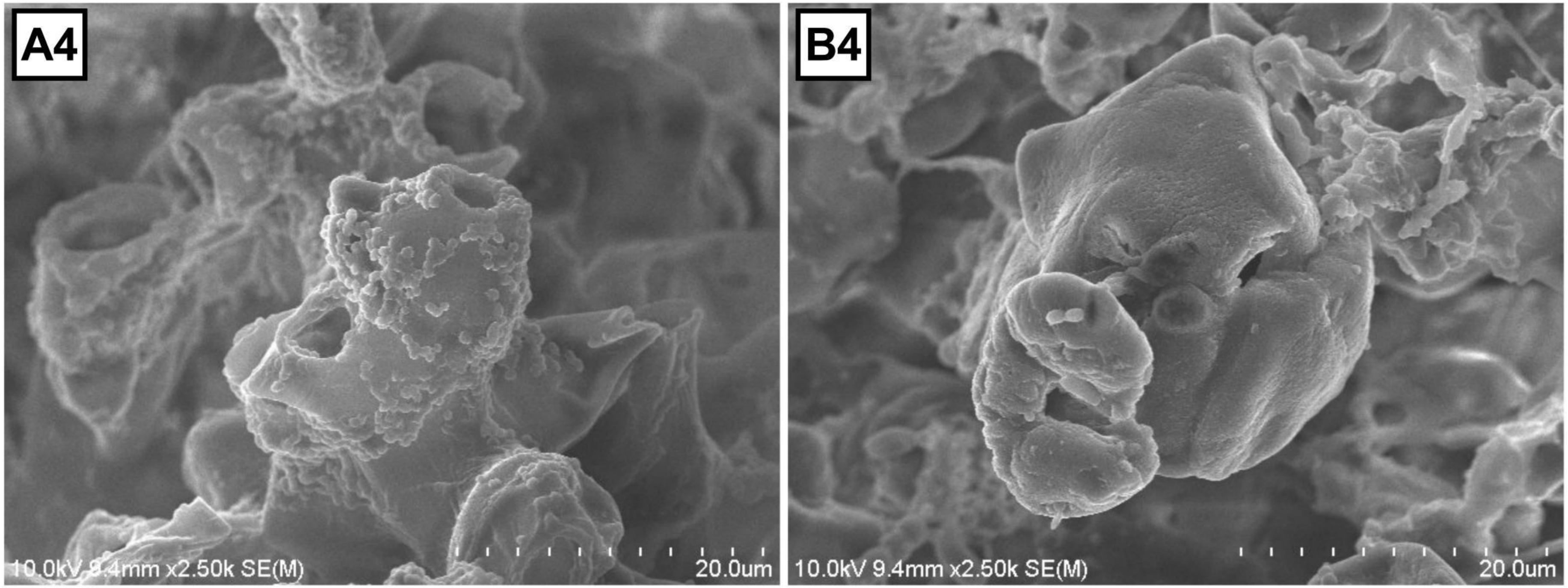
A4
10.0kV 9.4mm x2.50k SE(M)
20.0um
B4
10.0kV 9.4mm x2.50k SE(M)
20.0um

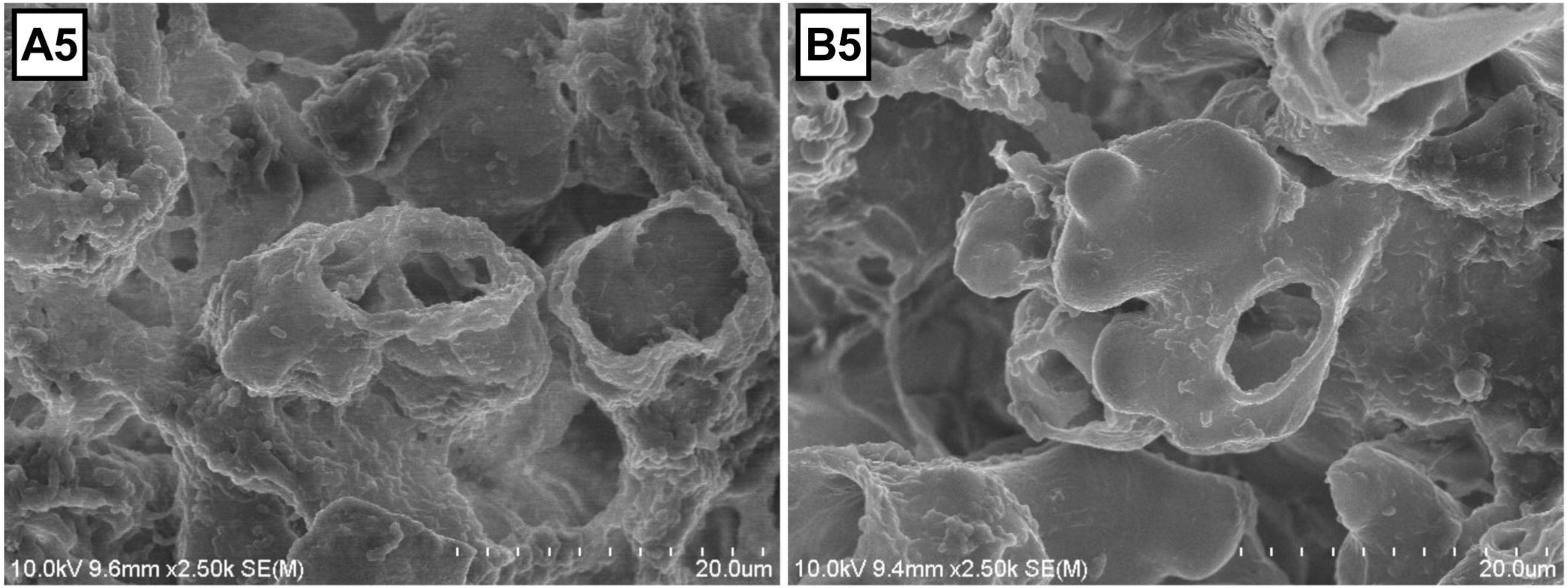
A5
10.0kV 9.6mm x2.50k SE(M)
20.0um
B5
10.0kV 9.4mm x2.50k SE(M)
20.0um

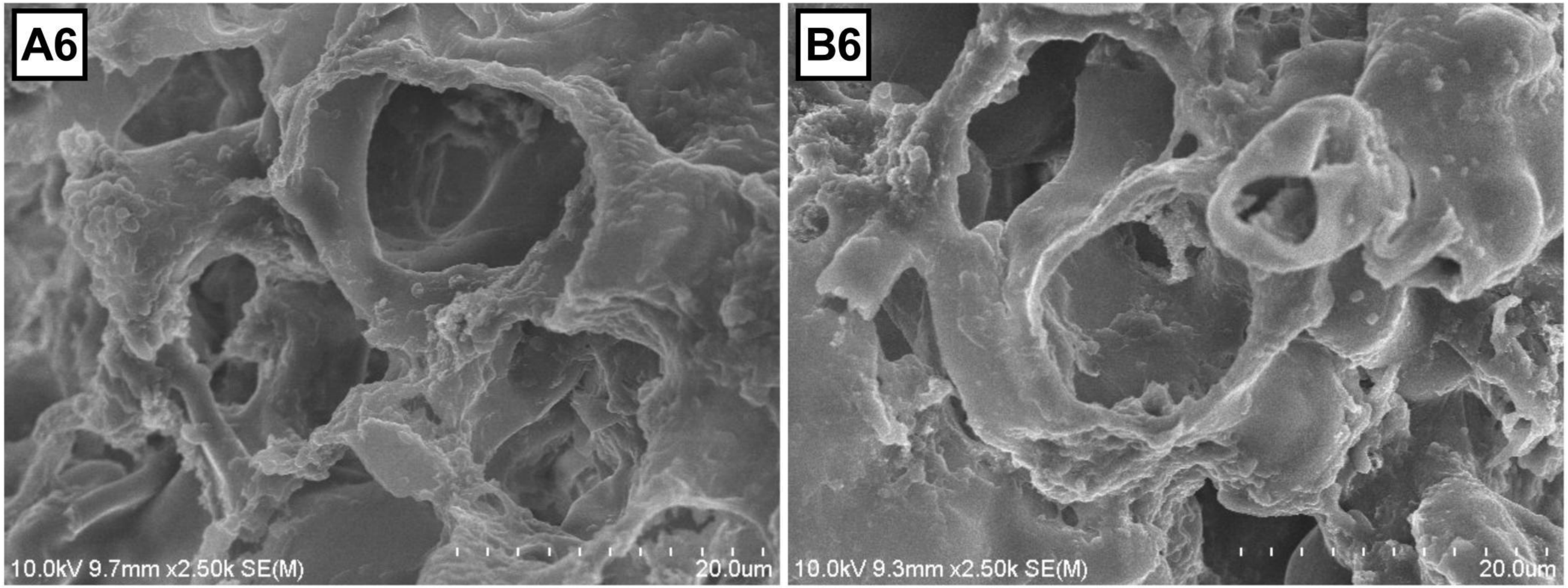
A6
10.0kV 9.7mm x2.50k SE(M)
20.0um
B6
10.0kV 9.3mm x2.50k SE(M)
20.0um

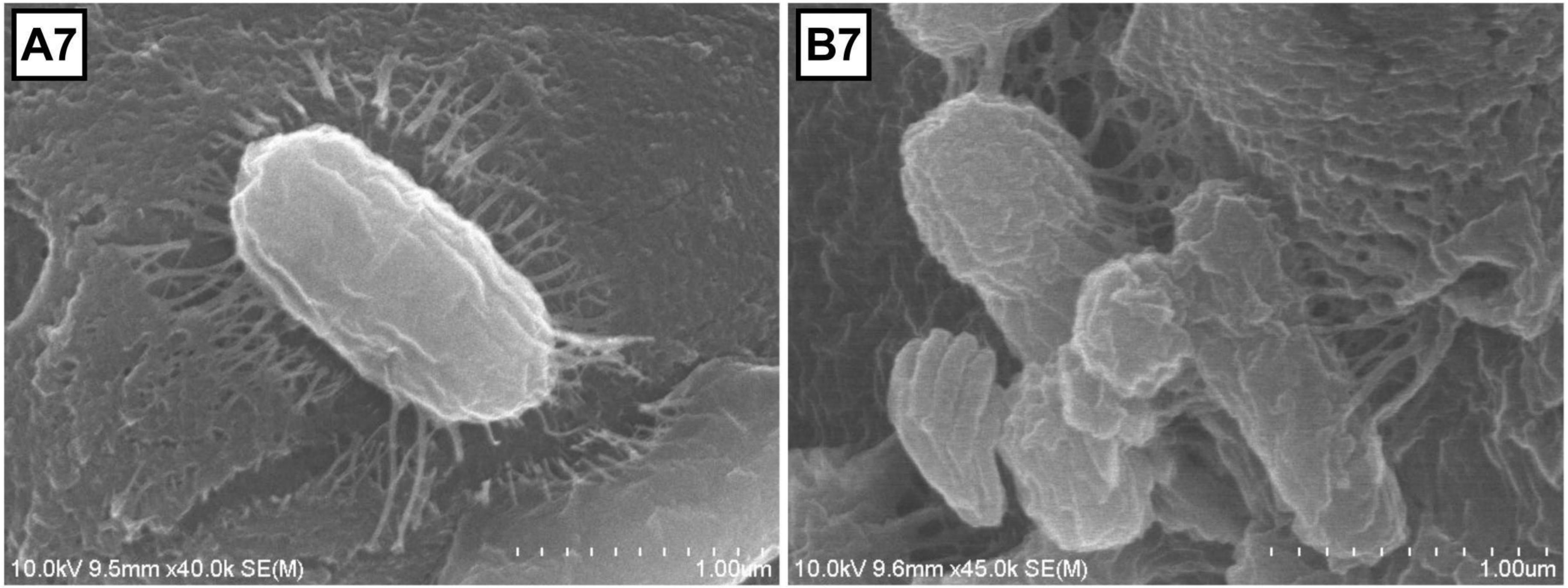
A7
10.0kV 9.5mm x40.0k SE(M)
1.00um
B7
10.0kV 9.6mm x45.0k SE(M)
1.00um
